# The Potential Use of Exosomes in Anti-Cancer Effect Induced by Polarized Macrophages

**DOI:** 10.3390/pharmaceutics15031024

**Published:** 2023-03-22

**Authors:** Chizumi Abe, Maharshi Bhaswant, Teruo Miyazawa, Taiki Miyazawa

**Affiliations:** New Industry Creation Hatchery Center (NICHe), Tohoku University, 6-6-10 Aramaki-aza-Aoba, Aoba-ku, Sendai 980-8579, Japan; chizumi.abe.e5@tohoku.ac.jp (C.A.); cmaharshi@gmail.com (M.B.); teruo.miyazawa.a7@tohoku.ac.jp (T.M.)

**Keywords:** antioxidant, cancer, exosome, immunotherapy, immunomodulation, inflammation, macrophage, macrophage polarization, polyphenols, tumor

## Abstract

The rapid development of aberrant cells outgrowing their normal bounds, which can subsequently infect other body parts and spread to other organs—a process known as metastasis—is one of the significant characteristics of cancer. The main reason why cancer patients die is because of widespread metastases. This abnormal cell proliferation varies in cancers of over a hundred types, and their response to treatment can vary substantially. Several anti-cancer drugs have been discovered to treat various tumors, yet they still have harmful side-effects. Finding novel, highly efficient targeted therapies based on modifications in the molecular biology of tumor cells is essential to reduce the indiscriminate destruction of healthy cells. Exosomes, an extracellular vesicle, are promising as a drug carrier for cancer therapy due to their good tolerance in the body. In addition, the tumor microenvironment is a potential target to regulate in cancer treatment. Therefore, macrophages are polarized toward M1 and M2 phenotypes, which are involved in cancer proliferation and are malignant. It is evident from recent studies that controlled macrophage polarization might contribute to cancer treatment, by the direct way of using miRNA. This review provides an insight into the potential use of exosomes to develop an ‘indirect’, more natural, and harmless cancer treatment through regulating macrophage polarization.

## 1. Introduction

Cancer is a global disease, from which nearly 10 million people died in 2020 [1]. Targeted cancer drugs approved by the Food and Drug Administration (FDA) in recent years have proven effective in blocking biological transduction pathways and/or specific cancer proteins to stimulate the immune system and induce apoptosis of cancer cells, or to deliver chemotherapeutic agents [2]. However, the paradoxical problem can occur where more potent anti-cancer drugs are more toxic to normal cells, leading to serious side-effects. It is also challenging to deliver drugs to target tumors due to the excellent ability of our biosystems to excrete strange molecules outside the body or destroy them. In the studies of nanoparticle drug delivery systems designed to deliver drugs to tumors, meta-analytic studies have reported that less than 1% of the administered dose accumulates in the target tumor [3]. Furthermore, it has been reported that even with the use of ligands to selectively target tumors, the tumor accumulation of modified nanoparticles remains at 0.7% of the administered dose, with 0.0014% of the nanoparticles interacting with cancer cells within the tumor [4]. Improving this low accumulation rate remains a major challenge in the field of cancer therapy.

Since the last decade, researchers have provided a lot of solutions such as drug modifications and environmental modifications, and developed drug delivery systems [5,6]. These cancer therapies are more effective and less harmful. Recently, exosomes have been gaining attention as a novel bio-functional carrier. These exosomes are a type of extracellular nanovesicle secreted from cells, working as a mediator of intercellular communication. Their inherent ability in transferring molecules to cells has attracted the interests of researchers to use them as the natural drug delivery systems for drug application, such as mRNAs, siRNAs, microRNAs (miRNAs), and small molecules [7]. In addition to this direct attack by drugs or chemotherapy as the means of cancer treatment, immunotherapy has also been proposed. Despite its long history, it was not until recent years that immunotherapy, which aims to eliminate malignant cells through natural immune defenses, has achieved significant advances in multiple forms of treatment, including cancer vaccines, adoptive cell transfer, and immune checkpoint inhibitors. Cancer immunotherapy is underpinned by the tumor infiltration of immune cells. Therefore, a better understanding of their profiles under a tumor microenvironment is essential and would shed light on the novel cancer therapies [8].

It is known that under a tumor microenvironment, macrophage polarization is skewed toward the M2 phenotype, which can be regulated via related cell signals. Apart from the promising role of the regulation of the tumor microenvironment in anticancer therapy and the high potential of exosomes as the natural transferer of biomolecules, the application of exosomes in the regulation of macrophage profiles in cancers is shown to a lesser extent. Therefore, this review aims to gain an insight into the potential application of exosomes as the regulator of macrophage polarization under a tumor microenvironment for novel anticancer therapy.

## 2. Macrophage Polarization

**Figure 1:**
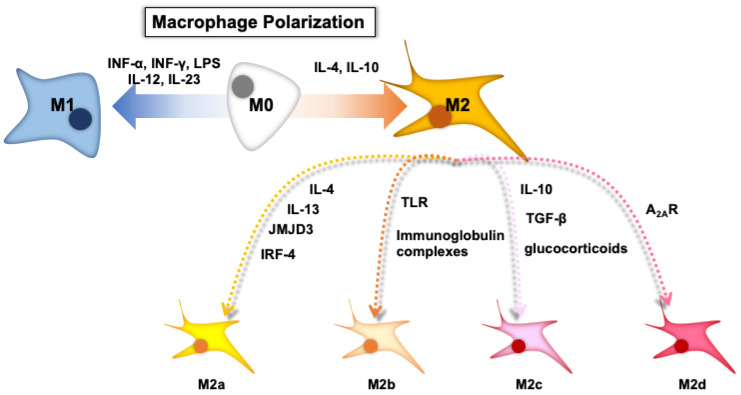
The depicted scheme of macrophage polarization. Note: Figure 1 was adapted and modified from a previous paper with permission from Elsevier [9].

Macrophages are immune cells that are responsible for innate immunity, which includes engulfing pathogens and promoting internal and external changes within the body. Macrophages are not homogeneous; they comprise three phenotypes: naïve macrophages (M0), pro-inflammatory macrophages (M1), and anti-inflammatory macrophages (M2) (Figure 1). M0 macrophages are widely distributed throughout the body and clean senescent/apoptotic cells and pathogens by phagocytosis, which are exposed to signaling molecules and released by lymphocytes or damaged tissues. M0 macrophages are stimulated by various cytokines and differentiate into M1 or M2 macrophages, which is called “macrophage polarization”. Toll-like receptor (TLR) ligands, lipopolysaccharides (LPSs), and interferon-γ (INF-γ) activate M1 macrophages, which have a high potential with antigen presentation and the expression of pro-inflammatory cytokines (interleukin (IL)-12, IL-23, and tumor necrosis factor (TNF)-α). IL-4 and IL-10 stimulate M0 macrophages to differentiate into M2 macrophages. Interestingly, M2 macrophages are further divided into four subtypes, M2a, M2b, M2c, and M2d, depending on the cytokines responsible for differentiation: M2a macrophages are induced by IL-4, IL-13, jumonji domain-containing-3 (JMJD3), and interferon regulatory factor (IRF)-4; M2b macrophages are induced by immunoglobulin complexes in combination with TLR agonists; M2c macrophages are induced by IL-10, transforming growth factor β (TGF-β), or glucocorticoids; M2d (described only in mice) can be induced by adenosine in pro-inflammatory M1 macrophages through activated adenosine 2A receptor (A_2A_R).

These characteristic formation changes of macrophages are used as therapeutic strategies. The exosomes from adipose-derived stem cells (ADSC-Exos) were loaded into a gelatin nanoparticle hydrogel (a good nanostructure hydrogel). These nanoscale molecular assemblies downregulated M1 marker (iNOS) expression but upregulated M2 marker expression (CD206), where ADSC-Exos are responsible for bone healing by macrophage polarization regulation via miR-451a [10]. In diabetic rats, infiltration and M1 macrophage polarization were enhanced in periodontal lesions with inflammation. Under high-glucose conditions, the reactive oxygen species (ROS) level was elevated with increasing macrophage polarization into M1, which was successfully reversed by N-acetyl cysteine, a ROS scavenger [11]. The neurotransmitter dopamine (DA) is closely related to inflammatory bowel disease, where DA regulates macrophage polarization via the DRD5 receptor highly expressed in colonic macrophages: DA–DRD5 signaling suppressed M1 but promoted M2 via the negative or positive regulation of NF-κB signaling and the CREB pathway [12]. The Yes-associated protein (a key transcription coactivator) drove macrophages toward M1 polarization while restricting M2 polarization, whose expression was different in two-type macrophages. Authors have suggested that this loop of YAP–macrophage polarization could be a crucial controller of diseases [13]. Food and its components also have the potential to regulate M1/M2 macrophage polarization. Previously, the essence of chicken has been reported for its increasing immunoglobulin level in serum and its activation of macrophage-like cells. Hydrolyzed chicken meat extract and its bioactive components were found to regulate macrophage polarization in RAW 264.7 cells comparably or more strongly than the essence of chicken, which additionally showed the significant amelioration of LPS-induced inflammation and oxidative stress [14]. Quercetin is a flavonoid present in vegetables such as onion. It is shown that, under excessive inflammatory conditions, this flavonoid inhibits M1 macrophage polarization via suppressed activation of the TLR/MyD88/NF-kB and IRF5 pathways to reduce the synthesis and release of inflammatory factors, as well as inhibits M2 macrophage polarization to reduce the excessive accumulation of extracellular matrix and interstitial fibrosis. These functions for moderating macrophage polarization changes imply that quercetin has therapeutic potential for kidney damage repair [15]. *Aronia melanocarpa* (*A. melanocarpa*), known as black chokeberry, is rich in anthocyanins, especially cyanidin-3-galactoside (C3G). Treatment with C3G from *A. melanocarpa* relieved inflammation while regulating macrophage polarization in pulmonary injury caused by PM10 (atmospheric particulate matter with an aerodynamic diameter ≤10 μm) [16]. The most common bowel inflammation “ulcerative colitis” is gradually spreading in developing countries, due to the inevitable side-effects of present medication, warranting novel and effective therapies. A flavonoid tiliroside, well reported for its anti-inflammatory biological activities, regulates macrophage polarization via metabolic pathway changes, contributing to alleviating clinical symptoms of the colitis [17]. Panax ginseng is an herb native to eastern Asia, which has been traditionally used as a medicinal preparation for various diseases over 2000 years. Ginsenosides are the major active component of the plant, among which ginsenoside Rg1 has been reported for a wide range of pharmacological effects such as anti-inflammatory, antioxidant, anti-cancer, anti-apoptotic, and neuroprotective effects. As a result of exploring the mechanism of ginsenoside Rg1 functions in recent studies, it alleviated a variety of diseases such as acute lung injury [18], cardiovascular and cerebral-vascular diseases [19], autoimmune encephalomyelitis [20], and ulcerative colitis by regulating M1/M2 macrophage polarization [21]. Other flavonoids are also reported to suppress inflammation via induced macrophage polarization from M1 into M2. Luteolin, a dietary flavonoid commonly contained in vegetables and fruits such as celery, green pepper, and apple peel, has been found to reduce the inflammatory reaction of activated macrophages as well as to show anti-tumor, antioxidant, anti-infection, immunomodulatory, and cardioprotective effects. A recent study elucidated that luteolin can also regulate macrophage polarization from proinflammatory (M1) into anti-inflammatory (M2) with downregulated p-STAT3 and upregulated p-STAT6 [22]. Soybean is a common food ingredient in Asian countries and shows potential beneficial effects on coronary heart diseases, atherosclerosis, type-2 diabetes, and gastrointestinal inflammation. Genistein is a bioactive component of soybeans and has also been found to modulate inflammation and reduce intestinal inflammation by binding estrogen receptors predominantly expressed in the gastrointestinal tract. In a recent study, genistein-affected T cells skewed macrophages from the M1 to M2 phenotype to reduce the severity of colitis in mice, induced by dextran sodium sulfate (DSS) [23]. Vitamin B, called niacin, is also reported to play a role in triggering and boosting anti-inflammatory immune responses and to possibly influence the cause of Parkinson’s disease, whose progression of inflammation is thought to be a central factor. Niacin skewed macrophage polarization from M1 to M2 via its receptor GPR109A in Parkinson’s disease subjects, where low-dosed niacin improved quality-of-life assessments in the subjects compared to high-dose treatment [24]. Furthermore, it has become clear that macrophage polarization is also induced by the Fenton reaction, a reaction in which iron reacts with hydrogen peroxide to produce hydroxyl radicals [25,26]. In tryptophan and α-ketoglutaric acid metabolism, as two essential effector pathways in macrophages, iron is involved in enzymatic steps to control metabolites’ macrophage effector functions. Therefore, currently, there has been a growing interest in elucidating the mechanism of redox reactions in cancer therapy.

## 3. Exosome

**Figure 2:**
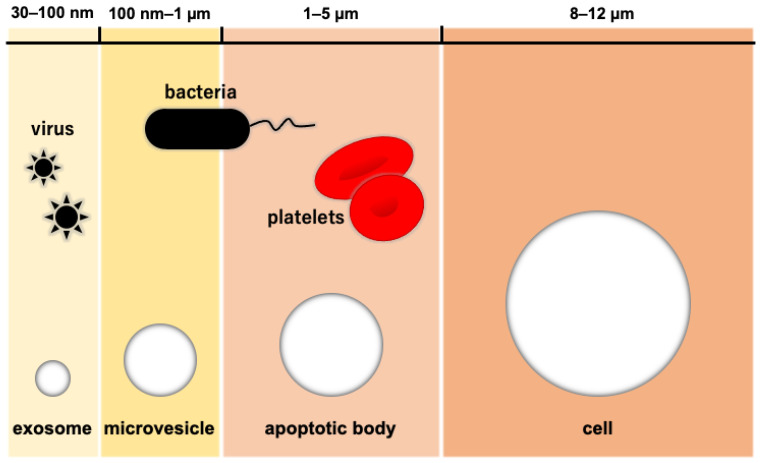
The relative size of extracellular vesicles. Note: Figure 2 was adapted and modified from a previous paper with permission from Elsevier [31].

Extracellular vesicles (EVs) are membrane vesicles containing various biological molecules such as mRNA, miRNA, DNA, proteins, and membrane receptors. EVs were thought to throw waste proteins and biomolecules until Valadi et al. found RNAs (mRNA and miRNA) in them and suggested their role as the mediator of intercellular communication [27]. Some of the EVs secreted from normal cells have been reported to potentially maintain their microenvironment to prevent cancer initiation [28,29], while tumor-derived EVs changed the cellular microenvironment to support tumor growth [30]. This evidence has shown potential roles of EVs in therapeutic treatment. Exosomes are small EVs with a diameter of 30–100 nm, which are the smallest compared with other EVs: micro-vesicles are 100 nm–1 µm in diameter and apoptotic bodies are 1–5 µm in diameter (Figure 2). Exosomes are generated from late endosomes followed by the formation of multi-vesicles, whose membrane is then sunken inward into intraluminal vesicles.

### 3.1. The Potential Use of Exosomes for Therapy

The latest database (ExoCarta) explains that exosomes comprise 9769 proteins, 3408 mRNAs, 2838 miRNAs, and 1116 lipids [32]. Thum reported exosomes that have a similar structure and composition to the cell membrane [33], contributing to their good tolerance in the body. The molecules contained in exosomes such as RNAs have been reported as tools for therapy [31,34]. Although lipid-like nanoparticles enhanced intracellular protein delivery, the transport of proteins into cells remains challenging due to many obstacles such as the low purification efficiency and tolerance against immune responses. Yim et al. reported a novel technique, an exosome-based delivery system EXPLORs (exosomes for protein loading via optically reversible protein–protein interactions), that does not require the isolation of recombinant proteins and reduces the immune response by a patient-customized delivery system [35]. These skeletal characteristics against the immune system are also helpful for the drug delivery to target tumors. Exosomes loaded with paclitaxel preferentially accumulated cancer cells and passed Pgp-mediated drug efflux even in multi-drug-resistant cancer cells, resulting in a significant inhibition of metastatic growth [36]. In order to improve the efficiency of exosome delivery to the targets, Gomari et al. expressed Designed Ankyrin Repeated Proteins (DARPins) as a specific ligand against HER2+ cells. As a result of assessments, doxorubicin loaded in exosomes exhibited cytotoxic effects comparable to its free form, and the exosomes were preferably taken by HER2+ cells. This indicates that exosomes expressed with DARPins are a promising means for selective treatment of HER2+ cell lines with less side-effects [37]. More details and reports on exosome-based drug delivery systems are well summarized in other reviews [38,39], and Wang et al. highlighted the challenges in this delivery system [40].

### 3.2. The Potential Use of Exosomes for Diagnostics of Cancers

Exosomes work in intercellular communication with their contents such as RNAs. Tumors and cancer cells also excrete exosomes in progression and change the microenvironment. More specifically, exosomes have information about themselves, which is also useful for diagnostics of diseases [41]. Nearly 50% of clinical trials are using exosomes toward biomarker applications [42]. Lung cancer is one of the leading causes of death worldwide, of which non-small cell lung cancer (NSCLC) is the major case accounting for around 85%. Based on the screening and quantification analysis, PLA2G10 mRNA and its corresponding protein were highly expressed in NSCLC patients and well associated with more aggressive characteristics and the overall survival of NSCLC. The combination of these factors showed a better detectivity than traditional tumor markers, indicating that they are a promising novel biomarker for NSCLC [43]. Prostate cancer (PCa) is one of the serious threats to men worldwide as a leading cause of death. Due to poor prognosis at the late stage of cancer, early diagnosis is warranted, but the present biomarker, a prostate-specific antigen, has low specificity. AMACR (an enzyme a-Methylacyl-CoA racemase) is known to be elevated in PCa tissue, where, in urine, exosomes are validated as a biomarker for clinical use to detect PCa at initial biopsy [44]. Biting et al. and Xinyi et al. explained in detail the advantages of exosomes as a liquid biopsy and their application as a potential complement [45,46].

Recently, novel techniques have been developed for the detection of exosome diagnostics. Although exosomes are predicted as a novel effective biomarker, absolute quantification or classification is difficult due to few exosomes to detect (in the concentration range of 10^−12^–10^−16^) produced in the early stage of cancer tumors. Recent digital quantification techniques, digital PCR and digital ELISA platforms, enable the sensitive detection of individual molecules with amplified signals by dividing samples uniformly into a large quantity of small compartments. Based on this, Liu et al. developed a droplet-based single-exosome-counting immunoassay approach for the digital quantification of exosomes, named droplet digital ExoELISA. This system enables the highly sensitive and specific quantification of exosomes (dynamic range, 5 log; limit of detection, 10 exosomes/µL), and was validated for use for the quantification of exosomes in a plasma sample [47]. Mass spectrometry (MS) is also a promising means for high-sensitivity identification and quantification of proteins. Yet, inefficient preparation with an elongated experimental time limits the use of MS-based proteomic analysis of exosomes. Buck et al. then developed a new simple preparation method with a photocleavable surfactant Azo, which is capable of extracting proteins difficult to solubilize, and stabilizes exosomal lysis and protein extraction. Combined with reversed-phase liquid chromatography coupled to a trapped ion mobility spectrometry (TIMS) quadrupole time-of-flight mass spectrometer, they annotated 91% of the identified proteins in the exosome/EVs databases including several important exosomal markers [48]. Of a large number of detection methods for exosomes, surface-enhanced Raman spectroscopy (SERS) seems useful for exosome biomarker profiling due to its high sensitivity and multiplex detection capability [49,50,51].

Furthermore, machine learning including artificial intelligence is being used as a tool to support the diagnostics. The SERS platform based on gold nanopyramids achieved single-molecule sensitivity [52] and successfully distinguished small EVs from different cells [53]. Then, Li et al. investigated the combination of the SERS spectra with machine learning by using small EVs from the tissue, blood, and saliva of patients suffering or not from gastric cancer (GC). As a result, the established alogism distinguished small EVs between GC patients and non-GC with an accuracy of 90, 85, and 72%, and the AUC (area under the curve) in the “leave-a-pair-of-samples out” validation was 0.96, 0.91, and 0.65 in tissue, blood, and saliva, respectively. This methodology is potentially applied as a non-invasive detection of diseases, not only GC [54]. A SERS-deep learning method for exosome diagnostics of cancers also showed high sensitivity and specificity even in stage I patients suffering from lung cancer [55]: the trained machine learning model classified normal and lung cancer cell lines with an accuracy of 95% and predicted even stage I lung cancer patients with an AUC of 0.910. Finally, a developed deep-learning-assisted SERS model achieved 100% accuracy for patients with different breast cancer subtypes who do not undergo standard surgery [56]. These significant advancements are hastening exosome diagnostics in early-stage cancer in the future.

## 4. Exosome-Induced Macrophage Polarization in Cancers

### 4.1. M1/M2 Macrophages in Cancers

Macrophages are stimulated by the condition of the tumor microenvironment and change their characteristics or macrophage polarization. Macrophages acquire the properties of the M2 macrophage to infiltrate tumor tissues and promote tumor progression and metastasis [57]. In recent reports, this traitorous macrophage contribution has been shown in breast cancer [58,59], gastric cancer [60], colon cancer [61,62], and lung cancer [63]. On the other hand, these results also suggest that the inhibition or suppression of M2 macrophages is a potential solution in cancer therapy [64]. Several factors have been reported as the inducer of M2 macrophage polarization (Table 1). Cathepsin K, a secretary protein upregulated by the imbalance of microbiota, accelerates M2 macrophage polarization via binding to toll-like receptor 4 and stimulates the secretion of cytokines IL10 and IL17, therefore promoting the invasion and metastasis of colorectal cancer [65]. SENP3, a type of protease associated in deconjugation following SUMOylation, seems to be related to M2 polarization, resulting in enhanced tumor proliferation and metastasis in patients suffering from breast cancer [66]. Other proteins are also reported for their role in cancer progression through enhanced M2 macrophage polarization [67,68]. Some gene expressions are also involved in cancer progression via M2 macrophage polarization [69,70]. Long non-coding (Lnc) RNAs are attracting attention by their unique feature: they are composed of ≥200 nucleotides and do not encode proteins but regulate multiple physiological processes, such as apoptosis, angiogenesis, and inflammation [71]. The regulatory roles of LncRNAs in cancer progression through M2 macrophage polarization have also been reported in cancers such as colorectal cancer, breast cancer, and prostate cancer [72,73,74]. Interestingly, an oncometabolite is involved in skewing the macrophage polarization into the M2 type. Lactic acid is produced from catalyzed pyruvic acid in aerobic glycolysis known as the Warburg effect. This oncometabolite contributes to a lower pH and promotes cell proliferation, in which additional lactate is reported to induce M2 macrophage polarization via the ERK/STAT3 signaling pathway in breast cancer [75] or MCT-HIF1α signaling in gastric cancer [76]. According to recent studies, downregulated paxillin [77] and cyclooxygenase [78], and enhanced pentraxin [79] might inhibit M2 macrophage polarization. Despite much evidence suggesting that M2 macrophage polarization increases cancer progression, there is very limited evidence that shows that suppression of M2 macrophage polarization reduces cancer progression, from which we hypothesize that the suppression of cancer progression is not necessarily involved with the suppression of M2 macrophage polarization but is involved with multiple other factors. Even so, the modulation of macrophage polarization, or the regulation of the balance, is certainly promising to change their effect. Apart from the role of M2 macrophages as the promoter for cancer progression, the M1 macrophage has been reported to contribute to anti-cancer systems (Table 1). Low-dose naltrexone, a morphinan-6-1,17-(cyclopropylmethyl)-4,5-epoxy-3,14-dihydroxy-, hydrochloride, has been known and used for its antitumor effects in several malignant tumors such as breast cancer and pancreatic cancer, where increased M1 macrophages and the activated Bax/Bcl-2/caspase-3/PARP pathway have lately been found to be associated [80]. TMP195, a selective class IIa HDAC (HDAC4, -5, -7, and -9) inhibitor, also shows an antitumor effect in colorectal cancer through M1 macrophage polarization [81]. As well as through activated signal pathways, exosomes seem a useful means in anticancer effects of M1 macrophages. Recently, it has been revealed that non-cording RNA carried by macrophage-derived exosomes can regulate immune systems [82]. The M1 macrophage is found to downregulate PDL1, a protein on a cell membrane regulating the T cell receptor, in gastric cancer cells via exosomal miRNA (miR)-16-5p [83]. In lung adenocarcinoma, miR-181a-5p in exosomes from M1 macrophages inhibited STK16 (Serine/threonine kinase 16) expression through ETS1 to regulate cell apoptosis [84]. LncRNA HOTTIP, the key molecules in the anticancer effect from M1 macrophage-derived exosomes, regulates the polarization of circulating monocytes into the M1 phenotype to suppress head and neck squamous cell carcinoma (HNSCC) progression [85]. Choo et al. also reported that exosome-mimetic nanovesicles derived from M1 macrophages are capable of repolarizing M2 macrophages toward M1 macrophages to enhance the antitumor efficacy of the immune checkpoint inhibitor therapy [86]. On the other hand, Baek et al. utilized the structure of exosomes [87]. The surface-modified macrophage-derived exosome-mimetic nanovesicle with PEG achieved a 7-fold higher blood circulation to the target tumor compared to bare. The gemcitabine, a familiar drug for blood cancer treatment, loaded with the M1 macrophage-derived exosome developed by Tang et al., showed significant cytotoxicity in MB49 cells and tumor suppression in a tumor-bearing mouse model [88]. Additionally, Zhao et al. loaded a drug deferasirox with gemcitabine into a M1-macrophage-derived exosome to develop a co-delivery system, resulting in a significant anticancer effect on the GEM-resistant PANC-1/GEM cells and 3D tumor spheroids [89]. The exosomes derived from M1 macrophages are also reported as a carrier for the efficient delivery of other drugs [90,91].

### 4.2. The Role of Tumor-Induced Exosomes in Macrophage Polarization

As described, exosomes are one of the means for intercellular communication and derived from tumor cells, have the potential to cause cancer proliferation, development, and the resulting metastasis. Gastric cancer is the third leading cause of cancer death worldwide, whose chronic progression of helicobacter pylori infection is the main factor. PD1 expression is enhanced by microbial infection, which has been found to be involved in skewing macrophage characteristics toward those of the M2 type. It was newly clarified that gastric-cancer-derived exosomes effectively induced PD1+ TAMs, inducing the favorable environment for gastric cancer progression [92]. In addition, lncRNA HLA complex group 18 (HCG18), regarded as an oncogene in various tumors, is also highly expressed in gastric cancer and accelerates tumor metastasis. The gene was found to increase in exosomes from gastric cancer cells and facilitate macrophage polarization to the M2 phenotype through a novel regulatory axis: lnRNA/mi-875-3p/KLF4 [93]. Gastric cancer also causes distant metastases, where the liver is the most important target organ through blood flow; in fact, 4–14% of patients with gastric cancer suffer from liver metastasis. Gastric-cancer-derived exosomal miR-519a-3p is found to be responsible for inducing macrophage polarization into the M2 type followed by accelerated angiogenesis and liver metastasis. Moreover, a potential use of exosomal miR-519a-3p as a novel biomarker for the diagnosis of gastric cancer-liver metastases is elucidated [94]. In the case of lung cancer, a leading cause of global cancer death, lung adenocarcinoma (LUAD) as the most prevalent type of NSCLC takes up nearly half of all cases. There is less of an understanding of the exosome interchanges between tumor cells and TAMs, resulting in inefficient therapy. Chen et al. elucidated that LUAD cells and TAMs crosstalk as follows: LUAD-cells-derived exosomal miR-19b-3p/RTPRD/STAT signaling leads to LINC00273 transcription and macrophage polarization to the M2 phenotype, which transmits LINC00273 to LUAD cells to induce LATS2/YAP/RBMX signaling activation, facilitating the package of miR-19b-3p in exosomes [95]. In addition, hypoxia is well known as a common phenomenon in the microenvironment, where more and more evidence has supported that it promotes cancer development by promoting cells to excrete exosomes and vesicles. Under the condition, hypoxic lung cancer cells were found to enhance the secretion of exosomes containing miR-21, which induces macrophage polarization toward the M2 phenotype by binding the 3′UTR of interferon-regulatory factor 1 (IRF1) followed by the downregulated expression of IRF1 in macrophages. This skewed macrophage polarization caused lung cancer proliferation [96]. Circular RNAs (circRNAs) and LncRNAs have been reported to be involved in tumor progression. CircRNAs are a class of endogenous non-coding RNAs originated from the back-splicing of 3′ and 5′ ends through exon or intron circularization. The up-regulated expression of circFARSA was observed, which, in exosomes derived from NSCLC cells, induced TAM polarization into the M2 phenotype through the PTEN/PI3K/AKT signaling pathway to promote NSCLC metastasis. Furthermore, an RNA-binding protein elF4A3 plays an important role in the biogenesis of circFARSA in NSCLC cells [97]. LncRNAs, a group of non-coding RNAs with a length >200 nucleotides, have been found to be involved in various biological processes, among which LINC00313 is a novel lncRNA reported to be expressed in several cancers and to control tumor growth and development. In a recent study, it was elucidated that LINC00313 promoted macrophage differentiation into the M2 phenotype with STAT6 up-regulated through sponging miR-135a-3p. This regulation of macrophage polarization and enhanced tumor progression by exosomal LINC00313 were evidenced in the mouse-xenograft models [98]. In other cancers, exosomes seem associated with macrophage polarization in leading to tumor progression. Pancreatic cancer is a malignant digestive cancer, whose 5-year overall survival is less than 5% mostly due to late diagnosis, aggressive tumor biology, resistance to chemotherapy, and the lack of the personalized treatment. Fibroblasts are known as the major components of cancer stroma, specifically to cancer-associated fibroblasts (CAFs). Fang et al. clarified that CAFs are intrinsically resistant to gemcitabine and exosomes derived from CAFs promoted the resistance of pancreatic cancers against gemcitabine, where miR-106b upregulated in CAFs responding to gemcitabine stimulation might be responsible [99]. Subsequently, they obtained the result that CAFs-derived conditioned medium facilitated macrophage polarization toward the M2 phenotype; there, the expression level of miRNA-320a most significantly increased in CAFs-derived exosomes. Upon further investigation on the detailed mechanism, it was found that miRNA-320a from CAFs-derived exosomes activated the PTEN/PI3Kγ signaling pathway to alter macrophage polarization [100]. Renal cell carcinoma (RCC) is the eighth leading malignancy worldwide, 85% of which is malignant and which accounts for 2–3% of all malignant diseases in adults. The major type, around 70–80%, of RCC is clear cell RCC (ccRCC) characterized by compact nests of tumor cells with the clear cytoplasm separated, which is difficult to cure by common therapies (radical nephrectomy, surgical nephrectomy, or medical treatment) due to its relapse or resistance. LncRNA AP000439.2 was found as a significant molecule in ccRCC-derived exosomes, where the lncRNA was highly expressed and promoted macrophage polarization into the M2 phenotype through the STAT3/NF-κB signaling pathway [101]. The worst and most significant characteristics of cancer are its excess proliferation and metastasis, causing a lower survival rate of patients, and thus detailed mechanisms and novel approaches are warranted to be clarified and developed. As described here, the remote control of macrophage polarization via exosomes is useful for tumor cells to make their surrounding microenvironment more comfortable to progress. Current reports on the roles of tumor-derived exosomes in the change in macrophage polarization are well reviewed with more detailed information by Baig et al. [102] and Xu et al. [103]

### 4.3. Anticancer Effects by Exosome-Induced Macrophage Polarization

As discussed in the above sections, (1) exosomes are one of the potential and attractive means for drug delivery, (2) macrophage polarization is involved in cancer progression or suppression, and (3) exosomes derived from tumor cells can regulate macrophage polarization to change their growing microenvironment. Considering the opposite effects coming from M1- and M2-type macrophages, it is important to balance them in anticancer therapy. The approaches that change the balance of macrophage phenotypes seem effective through dynamic regulation of immune systems [85,86]. As shown above, exosomes are a useful tool to regulate macrophage polarization in tumor cells, and then in current years, anticancer effects through the regulation of macrophage polarization by exosomes have been investigated [104] (Table 2). Cervical cancer is the fourth most common cancer among women worldwide and is difficult to diagnose in the early stage, where miR-423-3p was suggested as a potential modulator of the cancer progression. In in vitro and in vivo tests, the treatment with exosomal miR-423-3p successfully attenuated cervical cancer cell progression as well as tumor growth by inhibiting macrophage polarization to the M2 phenotype possibly through the CDK4-mediated IL-6/STAT3 pathway [105]. Human papillomavirus (HPV), especially HPV-16, has been considered as an etiological risk factor for HNSCC oncogenesis, and many studies have suggested that HPV possibly contributes to tumor biology and clinical characteristics including the response to radiation. In recent study, it was found that HPV had the potential to promote HNSCC cells to excrete exosomes, enriching miR-9 and downregulating PPARδ to induce M1 macrophage polarization, potentially resulting in the enhanced HNSCC radiosensitivity [106]. Moradi-Chaleshtori et al. investigated the conversion of macrophage polarization from the M2 to M1 phenotype by micro-RNA-loaded exosomes in vitro and in vivo. The MiR-33 family of miRNAs has been reported as a regulator of lipid metabolism with targeting genes involved in fatty acid metabolism, insulin signaling, and mitochondrial function. Exosomes from 4T1 breast cancer cells loaded with miR-33 showed an efficient delivery of the miRNA into macrophages, inducing macrophage polarization from the M2 to M1 phenotype. Furthermore, the incubation with conditioned media of treated macrophages inhibited the invasion and migration of tumor cells [107]. In addition, the further evaluation of the effect of miR-130 in modulating macrophage profiles assisted by exosome delivery suggests that miR-130 successfully converted macrophage polarization from the M2 to M1 phenotype, and macrophages treated with miR-130 containing exosomes reduced the proliferation, migration, and invasion of tumor cells [108]. According to these studies, they made miR-130 and miR-33 overexpressed in exosomes derived from MDA-MB-231 cells. The miRNAs-containing RNA successfully skewed macrophage polarization toward the M1 phenotype and suppressed the migration and invasion ability of cancer cells. Additionally, in BALB/c mice, injection of exosomes containing miR-130 or miR33, or both, decreased tumor volumes to values lower than those of the control [109]. As well as these “direct” approaches, or the way of inserting miRNA into exosomes, “indirect” approaches, or the way of inducing cells to release functional exosomes, have been interestingly investigated. Pigment-epithelium-derived factor (PEDF) is a monomeric 50 kDa glycoprotein and a member of the serpin superfamily of serine protease inhibitors, which is suggested by recent studies to function as an immune modulatory factor. The isolated exosomes secreted from MDA–MB-231 cells transfected with the PEDF-expressing vector significantly elevated the level of gene expression associated with M1 macrophages to reprogram the macrophages’ phenotype [110]. Epigallocatechin-3-gallate (EGCG), one of the most popular polyphenols in green tea, has been reported for its anti-tumorigenic activities, for example, inhibiting cell proliferation, invasion, and metastasis; inducing apoptosis and cell cycle arrest; suppressing angiogenesis. In a study, EGCG significantly inhibited tumor growth through inhibiting tumor-assisted macrophage infiltration and M2 polarization in vivo; moreover, ex vivo tests evidenced that EGCG-treated exosomes derived from 4T1 cells altered the macrophages’ polarization from the tumor-promoting M2 to the tumor-inhibiting M1 phenotype. It is evident that in 4T1 cells, EGCG up-regulated the level of exosomal RNAs, especially miR-16, which is responsible for inhibiting M2 macrophage polarization through IκB accumulation in tumor-assisted macrophages [111]. Protein tyrosine phosphatase receptor type O (PTPRO), belonging to the R3 subtype family of receptor-type protein phosphatase, has been reported to act as a tumor suppressor through its anti-inflammation and anti-immunity activity. Exosomal PTPRO induced the switch of macrophage polarization to the M1 phenotype mediated through the dephosphorylation, or inactivation, of STAT3 and STAT6 in inhibiting breast cancer invasion and migration [112]. Exosomes are promising as a carrier for drug or RNA delivery in cancer treatment, but more remains to be done in lowering the side-effects with higher specificity. Alternatively, it is also a potential strategy to change the target of exosomes from cancer to the factors regulating cancers. Although further studies on the effect of exogenous exosomes altering macrophage polarization on normal cells are required, it is an expected means to use the opposite effects coming from M1- and M2-type macrophages for treatment of cancer cells with much difference to normal cells.

## 5. Perspective and Conclusions

As cancer is one of most difficult diseases to treat worldwide, to date, significant efforts have been made in chemotherapy and immunotherapy. Among lots of promising means, these days, exosomes are attracting attention as a novel drug carrier and an efficient biomarker, in which advanced techniques are accelerating. On the other hand, secondary effects—serious side-effects—remain a problem for the treatment with drugs due to their lower selectivity. It has been clarified that the tumor microenvironment has unique characteristics, the regulation of which is regarded as a promising means of cancer treatment. As has been reviewed, macrophage polarization is possibly responsible for cancer proliferation through some proteins, but less seems clarified in their direct modulation. Although several agents targeting tumor-associated macrophages have been reported and available so far, we still have difficulties in translating them into clinical benefits due to an insufficient understanding of their complex immune systems [113]. Given the large number of research studies focusing on exosomes as the carrier working for direct transferring agents or molecules, the potential of exosomes in helping macrophage polarization should be investigated further for their better understanding. Clinical trials using exosomes and pre-clinical trials using animals are conducted worldwide [42]; however, several obstacles still remain: large-scale production, high-purity collection, standardized storage, efficient therapeutic effect, and so on [114]. It might take time to solve these challenges and limitations, which can be achieved by the continuous development in technological progress and cooperation between researchers. Furthermore, as well as direct approaches, relative and natural exosome-involved macrophage polarizations through the stimulation from adjacent or surrounding cells are also probably important to understand for novel cancer therapy. The recent studies suggest that a common dietary polyphenol EGCG regulates macrophage polarization via miRNAs that other cells secrete in response to EGCG stimulation, indicating a novel possible mechanism of the physiological regulation induced by dietary chemicals. In addition, plant-exosome-like nanovesicles and milk-derived extracellular vesicles are collecting attention for their physical stability and biosafety even though, here, an in-depth investigation of the roles of miRNA from various foods is still warranted to solve these challenges [115]. Taking this evidence into consideration, food intake can modulate immune systems to maintain healthy conditions by exosomes present in food or exosomal intercellular communication initiated from the stimulation by dietary compounds. A recent study administering turmeric-derived nanoparticles showed local anti-inflammatory effects in colitis model mice [116]. However, the exosomes derived from cigarette-smoke-extract-treated airway epithelial cells were found to progress chronic obstructive pulmonary disease via macrophage polarization [117]. As also mentioned in Section 2, macrophage polarization affects not only cancers but also other inflammation events. Additionally, many researchers showed only the “result” that “macrophages skewed to another types” by treatment with specific compounds or exosomes. Here, various factors involving macrophage polarization have been reported such as those shown in Figure 1 and by Sica et al. [118] (i.e., INFγ, TNFα, LPS, and others, for M1 skewing; IL-4, IL-13, IL-10, and others, for M2 skewing). However, the specific and/or essential factors leading to macrophage polarization are not clarified: we mentioned in Section 3.2 that there is little direct evidence showing that suppressed M2 macrophages inhibit cancer proliferation. An in-depth understanding on macrophage polarization is warranted for cancer therapy to control the complicated tumor microenvironment. Further investigation on the roles of exosomes derived from various origins such as daily food in regulating macrophage polarization would be a step toward the development of more natural and harmless cancer therapies in the future.

## Figures and Tables

**Table 1:** Molecules involved in macrophage polarization.

Molecules	Tested Cancer	Polarization Skewing	Feature	Ref.
Cathepsin K	colorectal cancer	M2	promoted tumor metastasis	[65]
SENP3	breast cancer	M2	promoted cancer progression	[66]
B7-H3	ovarian cancer	M2	promoted cancer progression	[67]
DNMT1	lung cancer	M2	promoted cancer progression	[68]
Kdm6a	bladder cancer	M2	caused bladder cancer	[69]
Fstl3	gastric cancer	M2	macrophage infiltration	[70]
LncRNA MIR155HG	colorectal cancer	M2	drug resistance of cancer cells	[72]
LncRNA GNAS-AS1	breast cancer	M2	promoted cancer progression	[73]
LINC00467	prostate cancer	M2	promoted cancer progression	[74]
Lactic acid	breast cancer	M2	–	[75]
	gastric cancer	M2	–	[76]
Low-dose naltrexone	malignant tumors	M1	anti-tumor effect	[80]
TMP195	colorectal cancer	M1	anti-tumor effect	[81]
miR-16-5p (M1 macrophage derivative)	gastric cancer	–	regulated T cells	[83]
miR-181a-5p (M1 macrophage derivative)	lung adenocarcinoma	–	regulated apoptosis	[84]
LncRNA HOTTIP (M1 macrophage derivative)	head and neck squamous cell carcinoma	–	anti-cancer effect	[85]

**Table 2:** Exosomal molecules responsible for anti-cancer effects via macrophage polarization.

Types of Cancer	Cargo	Exosomes Derived from	Feature	Ref.
Cervical cancer	miR-423-3p	HeLa cell	attenuated cancer cell progression	[105]
HNSCC oncogenesis	miR-9	HPV + HNSCC cell	enhanced radiosensitivity	[106]
Breast cancer	miR-33	4T1 breast cancer cells	inhibited the invasion and migration	[107]
	miR-130	4T1 breast cancer cells	reduced proliferation, migration, and invasion	[108]
	miR-130 and miR-33	MDA-MB-231 cells	lowered tumor volumes	[109]

## Data Availability

Not applicable.

## Abbreviations

AMACR	an enzyme a-Methylacyl-CoA racemase
M2	anti-inflammatory macrophage
AUC	area under the curve
CAFs	cancer-associated fibroblasts
circRNAs	circular RNAs
ccRCC	clear cell RCC
C3G	cyanidin-3-galactoside
DA	dopamine
EGCG	epigallocatechin-3-gallate
DSS	dextran sodium sulfate
ADSC-Exos	exosome from adipose-derived stem cells
EXPLORs	exosomes for protein loading via optically reversible protein–protein interactions
EVs	extracellular vesicles
GC	gastric cancer
HNSCC	head and neck squamous cell carcinoma
HCG18	HLA complex group 18
IRF	interferon regulatory factor
IRF1	interferon-regulatory factor 1
INF-γ	interferon-γ
IL	Interleukin
JMJD3	jumonji domain-containing-3
LPS	Lipopolysaccharides
Lnc RNA	long non-cording RNA
LUAD	lung adenocarcinoma
miRNAs	microRNAs
M0	naïve macrophage
NSCLC	non-small cell lung cancer
PEDF	pigment epithelium-derived factor
M1	pro-inflammatory macrophage
PCa	prostate cancer
PTPRO	protein tyrosine phosphatase receptor type O
ROS	reactive oxygen species
RCC	renal cell carcinoma
STK16	serine/threonine kinase 16
SERS	surface-enhanced Raman spectroscopy
TLR	toll-like receptor
TGF-β	transforming growth factor β
TIMS	trapped ion mobility spectrometry
TNF	tumor necrosis factor
